# An Integrated System Biology Approach Yields Drug Repositioning Candidates for the Treatment of Heart Failure

**DOI:** 10.3389/fgene.2019.00916

**Published:** 2019-09-25

**Authors:** Guodong Yang, Aiqun Ma, Zhaohui S. Qin

**Affiliations:** ^1^Department of Cardiovascular Medicine, First Affiliated Hospital of Xi’an Jiaotong University, Xi’an, China; ^2^Department of Biostatistics and Bioinformatics, Emory University, Atlanta, GA, United States

**Keywords:** heart failure, drug repositioning, gene signature, systems biology, connectivity map

## Abstract

Identifying effective pharmacological treatments for heart failure (HF) patients remains critically important. Given that the development of drugs de novo is expensive and time consuming, drug repositioning has become an increasingly important branch. In the present study, we propose a two-step drug repositioning pipeline and investigate the novel therapeutic effects of existing drugs approved by the US Food and Drug Administration to discover potential therapeutic drugs for HF. In the first step, we compared the gene expression pattern of HF patients with drug-induced gene expression profiles to obtain preliminary candidates. In the second step, we performed a systems biology approach based on the known protein–protein interaction information and targets of drugs to narrow down preliminary candidates to obtain final candidates. Drug set enrichment analysis and literature search were applied to assess the performance of our repositioning approach. We also constructed a mode of action network for each candidate and performed pathway analysis for each candidate using genes contained in their mode of action network to uncover pathways that potentially reflect the mechanisms of candidates’ therapeutic efficacy to HF. We discovered numerous preliminary candidates, some of which are used in clinical practice and supported by the literature. The final candidates contained nearly all of the preliminary candidates supported by previous studies. Drug set enrichment analysis and literature search support the validity of our repositioning approach. The mode of action network for each candidate not only displayed the underlying mechanisms of drug efficacy but also uncovered potential biomarkers and therapeutic targets for HF. Our two-step drug repositioning approach is efficient to find candidates with potential therapeutic efficiency to HF supported by the literature and might be of particular use in the discovery of novel effective pharmacological therapies for HF.

## Introduction

Heart failure (HF) remains a worsening global public health problem with high mortality (Mosterd et al., 2001; Askoxylakis et al., 2010). Although the use of neuro-hormonal interventions in the forefront of HF treatment has substantially reduced mortality and improved the prognosis, morbidity and mortality remain unacceptably high (Mosterd et al., 2001; Askoxylakis et al., 2010; Lewis et al., 2017). The mortality of HF patients within 5 years is >50%, higher than most malignancies (Askoxylakis et al., 2010). Thus, there is an urgent need to identify new effective therapeutic treatments functioning through different mechanisms compared to existing treatments in order to improve the prognoses of HF patients.

However, developing drugs *de novo* is expensive and time consuming. To bring a new drug to market, it takes an average of 10 years and at least $1 billion in research and development (Xue et al., 2018). Given the time and cost of developing novel drugs, drug repositioning has become an increasingly important strategy, and many different computational methods have been developed to facilitate this process (Lamb et al., 2006; March-Vila et al., 2017; Subramanian et al., 2017; Peyvandipour et al., 2018). Drug repositioning aims to identify new applications for existing drugs to treat different diseases (Novac, 2013), which eliminates the need for preclinical development and optimization, hence saving effort, expense, and avoiding the high rate of failures that are typically associated with the new drug discovery process.

Classical drug repositioning methods include ligand-based approaches (Keiser et al., 2009; Liu et al., 2010; Vasudevan et al., 2012; Anighoro et al., 2015; Iwata et al., 2015; Mervin et al., 2015; Sawada et al., 2015) and structure-based approaches (Manning et al., 2002; Zhang et al., 2004; Kinnings et al., 2009; Defranchi et al., 2010; Jalencas and Mestres, 2013; Bowman et al., 2015; Hall et al., 2015; Ehrt et al., 2016). The concept for ligand-based approaches is that similar compounds are likely to have similar biological properties. Structure-based approaches are based on the concept that proteins with similar structures tend to have similar functions and to bind similar compounds. These two approaches are based on existing information regarding drugs and proteins. Drugs identified using these two types of methods share the same mechanisms as the template drugs. For a certain disease, if the template drugs have already been used as treatments, these approaches will play a limited role, and thus, other types of drug repositioning approaches able to find therapeutic drugs with different mechanisms compared to the existing treatments are required.

As we enter the “big data” era, given the generation and wide access of disease and drug-induced gene expression profiling data, such as the Library of Integrated Network-based Cellular Signatures (LINCS) L1000 dataset (Subramanian et al., 2017) as an eminent repository for pharmacogenomics and the expansion of the Connectivity Map (Cmap) (Lamb et al., 2006), noval, data-driven approaches have been developed to explore the possibility of drug repositioning through intelligent data mining to find drugs that function through different mechanisms compared to existing treatments (Lamb et al., 2006; Hu and Agarwal, 2009; Chang et al., 2010; Jin et al., 2012; Iskar et al., 2013; Subramanian et al., 2017; Peyvandipour et al., 2018). The first class of approaches is based on transcriptomic data. By comparing the gene expression patterns of diseases and drugs, therapeutic relationships between known drugs and new disease indications can be established (Lamb et al., 2006; Subramanian et al., 2017). Another class of methods aims at organizing the relationships among disease-related genes and drug targets in the form of a network to visualize the potential therapeutic efficiency of a drug (Peyvandipour et al., 2018). The approaches based on data mining can be used to find drugs that function through different mechanisms compared to existing treatments, but how to increase the likelihood of identifying actual disease-related candidate drugs remains a critical point.

**Figure 1:**
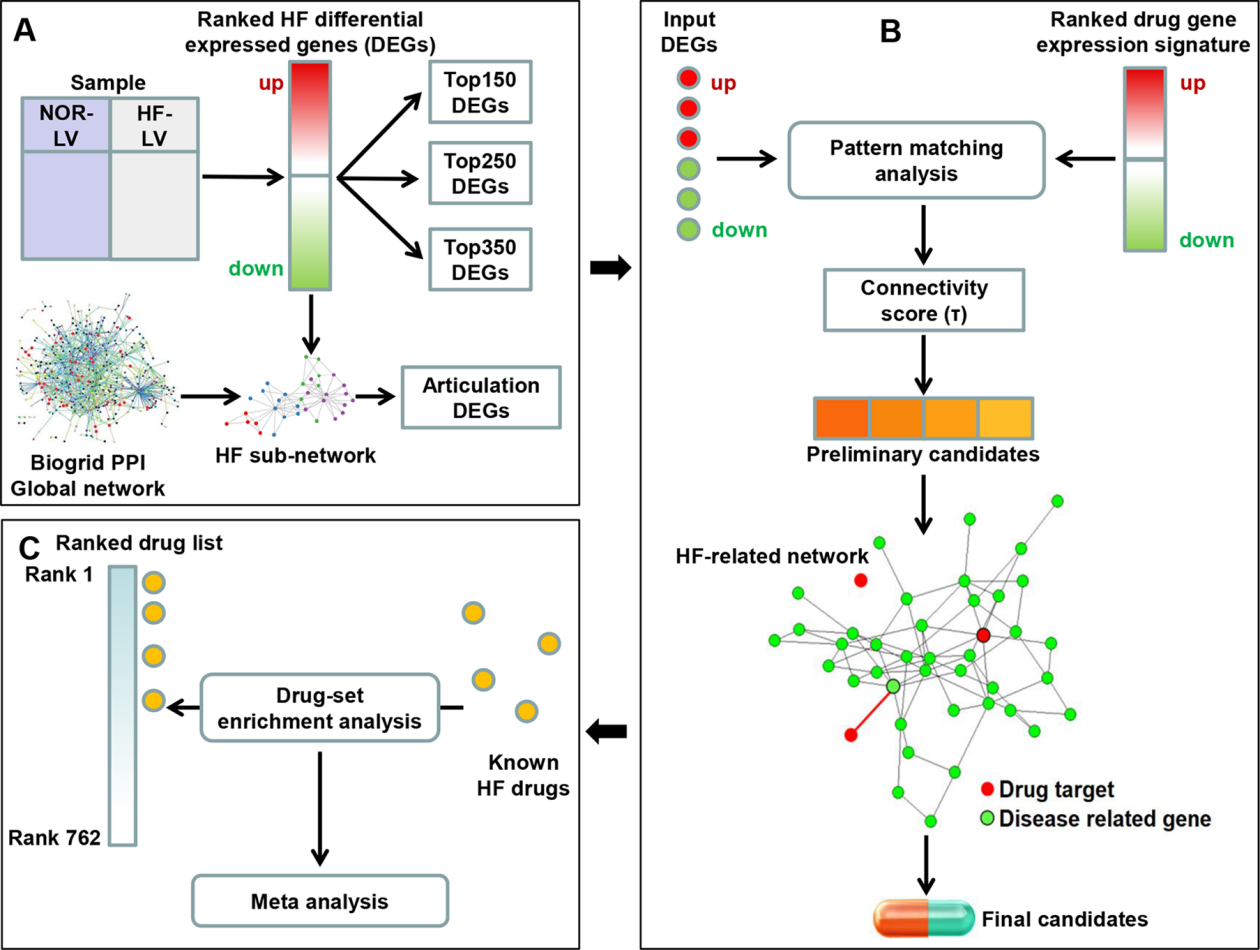
Framework overview. **(A)** We compared the gene expression profile of heart failure (HF) and normal human left ventricular (LV) tissues to obtain the differential expression genes (DEGs). The K-most top (up- and downregulated) DEGs were obtained according to the fold change values. We then extracted a HF subnetwork from the global protein–protein interaction network using all DEGs. Articulation DEGs of the subnetwork were extracted as inputs to perform subsequent drug repositioning analysis along with top *K* genes (*K* was set as 150, 250 and 350). **(B)** A pattern matching analysis was applied on input HF DEGs and drug-induced gene expression signatures. Connectivity score (τ) was calculated to assess the reversal of gene expression patterns and to select the preliminary candidates. Then, the interaction between the targets of preliminary candidates and HF-related network was investigated. Preliminary candidates whose targets located in the HF-related network were selected as final candidates. **(C)** Drug-set enrichment analysis was applied to determine whether known HF drugs are located higher in the whole drug list ranked by τ. Results across all datasets were aggregated using meta-analysis.

In the present study, we constructed a two-step drug repositioning pipeline by combining a transcriptome-based approach and a network-based approach to find candidate drugs approved by the US Food and Drug Administration (FDA) for HF. Drug-set enrichment analysis and literature search were applied to validate our approach. We also constructed a mode of action (MOA) network for each candidate to investigate their potential MOA. Then, a pathway analysis was performed using genes contained in the MOA network of each candidate to uncover the potential mechanisms of candidate’s therapeutic efficacy to HF (**Figure 1**).

## Materials and Methods

### Date Sources

#### HF Gene Expression Data

Gene expression data comparing HF and healthy human left ventricular tissues with a minimum sample size of 10 were downloaded from Gene Expression Omnibus (GEO). Twenty-one datasets, representing two HF subtypes: dilated cardiomyopathy (DCM) and ischemic cardiomyopathy (ISCM), satisfied the selection criteria (**Supplemental Tables 1** and **2**).

#### Drug-Induced Gene Expression Data and Drug Target Data

Drug-induced gene expression profiles were derived from the Library of Integrated Network-Based Cellular Signatures (LINCS) L1000 dataset (Subramanian et al., 2017), an eminent pharmacogenomics repository used for diverse purposes (Wang et al., 2016; Musa et al., 2018; Musa et al., 2019). The data are available in GEO (GSE92742), which contains transcriptional gene expression data from cultured human cells exposed to over 20,000 small molecules including most of the FDA-approved drugs. The LINCS L1000 dataset compares two groups of samples (control vs. treatment) and provides differentially expressed genes (DEGs) in terms of *z*-score signatures. Drug targets were retrieved from the database DrugBank, which combines detailed drug data with comprehensive drug target information (Wishart et al., 2018). Overall, we extracted 762 FDA-approved drugs with both gene expression signatures and known targets to perform the subsequent drug repositioning analysis.

#### Protein–Protein Interaction Network

Protein–protein interaction (PPI) network information was derived from the BioGRID database, one of the largest repositories of experimentally verified PPIs (Chatr-Aryamontri et al., 2017). We extracted all genetic and protein interactions for humans to build the global PPI network. In the global PPI network, each node is a gene, and the edges represent PPIs between nodes.

#### Known HF-Related Genes

HF-related genes were retrieved from the Comparative Toxicogenomics Database, a robust and publicly available database containing gene–disease association information (Mattingly et al., 2006). Genes associated to systolic HF were extracted for underlying analysis.

### Data Analysis

#### Detection of DEGs

For HF gene expression data collected from microarray, R/Bioconductor package Limma was applied (Smyth, 2004); for HF gene expression data collected from RNAseq, R/Bioconductor package edgeR was used (Robinson and Smyth, 2007). The definition of DEGs was set to an adjusted *P* value (<0.05). We used the top (K-most up- and downregulated) DEGs according to the fold change to represent the most significant and important DEGs of HF and perform the subsequent drug repositioning analysis. Different values of *K* (150, 250, and 350) were attempted.

#### Articulation DEGs Selection

We constructed a global network based on all BioGRID PPIs. For each HF-related dataset, we extracted a subgraph from the global network using the function *induced_subgraph* built in the R/Bioconductor package *igraph*. The nodes of the subgraph represent all DEGs, and the edges of the subgraph refer to the experimentally verified PPIs among the DEGs. Then, the articulation points of each subgraph were defined as the articulation DEGs using the function *articulation_points* built in the R/Bioconductor package *igraph*. Articulation points are key vertices in a network, whose removal increases the number of connected components in a graph. The way of finding articulation DEGs is to remove all nodes of a network one by one, and the nodes whose removal results in disconnected graph are selected as articulation DEGs. The articulation DEGs were considered as another subset of the most significant and important DEGs of HF besides top DEGs and were used to conduct the subsequent drug repositioning analysis.

### Drug Repositioning Analysis

#### Step 1: Obtain Preliminary Candidates Using a Transcriptome-Based Approach

In this step, we follow the same strategy as in Cmap (Lamb et al., 2006; Subramanian et al., 2017).

##### Step 1a. Calculate the Weighted Connectivity Score

First, we conducted a pattern-matching analysis (Lamb et al., 2006) to evaluate the reversal of gene expression patterns between HF DEGs and drug-induced gene signature. HF DEGs comes from one of the four kinds of HF gene expression patterns, that is top 150 DEGs, top 250 DEGs, top 350 DEGs, and articulation DEGs. Drug-induced gene signature refers to the gene expression profile of a certain cell type treated by drugs. For the HF DEGs, we compared the sorted upregulated and sorted downregulated DEGs, respectively, with drug-induced gene signatures sorted by *z*-scores of which upregulated genes are located at the top and downregulated genes are located at the bottom. For a given pair of HF DEGs and drug-induced gene signature, if upregulated HF DEGs enrich at the bottom of the sorted drug-induced gene signature and downregulated DEGs of HF enrich at the top of the sorted drug-induced gene signature, they are assumed to have the reversed gene expression patterns and thus have therapeutic potential. The Kolmogorov–Smirnov (KS) statistic presented in the original Cmap paper (Lamb et al., 2006) was used to quantitatively assess the reversal of gene expression patterns.

$$KS=\left\{\begin{array}{ll} a=\underset{1\;\leq \;j\;\leq \;t}{\max}\;\left[\frac{j}{t}-\frac{V\;(j)}{n}\right]\; & ,\;a>b\\ -b=-\underset{1\;\leq \;j\;\leq \;t}{\max}\;\left[\frac{V\;(j)}{n}\;-\frac{\left(j-1\right)}{t}\right] & ,\;b>a \end{array}\right\}$$

where *KS* is divided into *KS*_up_ and *KS*_down_ referring to the enrichment score for the sorted upregulated and sorted downregulated genes in a list of HF DEGs, respectively. The sorted upregulated HF DEGs were compared with drug-induced gene signatures sorted by *z*-scores to get *KS*_up_, and the sorted downregulated HF DEGs were compared with drug-induced gene signatures sorted by *z*-scores to get *KS*_down_. Let *n* be the total number of genes in an ordered drug-induced gene signature and *t* be the number of the up- or downregulated genes in a list of HF DEGs. *V(j)* is the position of gene *j* in the ordered drug-induced gene signature. Gene *j* is any gene in the sorted up- or downregulated gene list of HF DEGs, where *j* = 1, 2, …, *t*.

The weighted connectivity score was calculated based on the *KS* statistic as in the original Cmap paper (Lamb et al., 2006). For a given HF DEGs (*r*) and drug-induced gene signature (*q*), the weighted connectivity score *W*_q,r_ is calculated as follows:

$$Wq,r=\left\{ \begin{array}{c} KS_{up}-KS_{down},if\;\mathrm{sgn}(KS_{up})\neq \mathrm{sgn}(KS_{down})\\ 0,\;otherwise \end{array}\right.$$

where *KS*_up_ is the enrichment of upregulated HF DEGs in the sorted drug-induced signature, and *KS*_down_ is the enrichment of downregulated HF DEGs in the sorted drug-induced signature. The sign of *KS* will be negative if the upregulated DEGs of HF enrich to the bottom of the drug-induced signature or the down-regulated HF DEGs enrich to the top of the drug-induced signature. Otherwise, the sign of *KS* will be positive. Zero is assigned for cases where *KS*_up_ and *KS*_down_ are the same sign.

##### Step 1b. Calculate the Normalized Connectivity Score

Since the drugs’ perturbations are profiled in several cell lines, to each candidate, we calculated the weighted connectivity score for every pair of drug-induced gene signature and HF DEGs and then normalized the vector of weighted connectivity scores following the strategy of Cmap to allow for comparing across different cell types and different drug types (Subramanian et al., 2017). To a given vector of the weighted connectivity scores (*W*), we normalized *W* using the formula described previously (Subramanian et al., 2017) to obtain normalized connectivity scores (NCS):

$$NCS_{c,t}=\left\{\begin{array}{l} W_{c,t}/\mu_{c,t}^{+}\;,\;if\;\mathrm{sgn}(W_{c,t})>0\\ W_{c,t}/\mu_{c,t}^{-}\;,\;otherwise \end{array}\right\}$$

where *NCS_c,t_* is the normalized connectivity score for a certain pair of cell type (*c*) and perturbation type (*t*), *W_c,t_* is the weighted connectivity score, and *μ* ^+^ *_c,t_* and *μ^−^_c,t_* are the separately evaluated signed means of the positive and negative values of the weighted connectivity scores.

##### Step 1c. Calculate the Terminal Connectivity Score (τ)

While NCS can be used to make meaningful comparisons of the reversal between drug-induced signatures and disease-related gene sets, it is also useful to assess if the reversal between different combinations of drug-induced signature and disease-related gene set are significantly different. Tau (*τ*) (Subramanian et al., 2017) was used to compare the reversal of a certain pair of HF and drug with other pairs.*τ* was calculated as follows:

$$\tau_{q,r}=sgn(NCS_{q,r})\frac{100}{N}\sum_{i=1}^{N}\left[|NCS_{i,r}|\;<\;|NCS_{q,r}|\right]$$

where *NCS_q,r_* is the normalized connectivity score for a certain pair of drug-induced signature (*q*) and disease-related gene set (*r*). *NCS_i,r_* is the normalized connectivity score for the disease-related gene set (*r*) and any other drug-induced signature (*i*). *N* is the total number of drug-induced signatures. *τ* ranges from -100 to 100, and a | *τ* | of 90 indicates that to a certain drug-induced signature, 10% of the total drug-induced signatures showed stronger connectivity with the disease-related gene set than the certain drug-induced signature. The negative sign of *τ* refers to reversed gene expression patterns of drug and disease, while a positive sign indicates nonreversed gene expression patterns.

As each drug was profiled using several cell lines in Cmap, to find drugs most likely having therapeutic potential to HF, we used the minimum *τ* (the most negative *τ*) of all the cell lines as the connectivity score. The *τ* of the four lists of HF DEGs (top 150 genes, top 250 genes, top 350 genes, and articulation DEGs) used as inputs were averaged as the final score. For each dataset, we chose the top 5% (38) drugs with the most negative *τ* values as the candidate drugs. We then combined the top 5% drugs of all datasets and chose the 38 most common drugs as the preliminary candidate drugs.

#### Step 2: Narrow Down Preliminary Candidates Applying a Network-Based Approach

First, we built a HF-related network. To obtain representative DEGs of HF to build the network, we obtained the DEGs of each dataset and chose the most common DEGs across the majority of all datasets (three or more) as the final DEGs. Using the final DEGs, we extracted the HF-related network from the global network established previously. Then, we analyzed the relationship between candidates’ targets and the HF-related network. There are generally three different types of relationship between drug targets and disease network: (1) drug targets have no interactions with the disease network, (2) drug targets do not belong to the disease network but can interact with the nodes, and (3) drug targets are contained in the disease network. In our study, preliminary candidate drugs were selected as final candidate drugs, only if some of their targets are located in the HF-related network.

### Drug-Set Enrichment Analysis of Known HF Drugs

To assess the performance of our drug repositioning method, we performed a drug-set enrichment analysis as previously described (So et al., 2017) to determine if known HF drugs are ranked significantly higher than they would have been by chance in the whole drug list ranked by *τ*. We performed the enrichment analysis for each dataset separately. First, we ranked drug list of each HF dataset by *τ*. Then, we did rank-sum test to compare the difference of ranks of drugs being used in clinical practice with the ranks of the rest drugs. Finally, we aggregated the results across all datasets using meta-analysis. We applied three different methods to summarize the results: the inverse-variance method, Fisher’s method, and Tippett’s minimum P method (So et al., 2017).

### Manual Curation of the Final Candidate Drugs

To assess the results obtained from our method, we also performed a literature search with predefined search strategies to locate evidence of the therapeutic potential of candidate drugs for HF. Details regarding the query are as follows: DCM, Drug_name AND (DCM OR heart failure OR dilated cardiomyopathy OR dilated cardiomyopathies); ISCM, Drug_name AND (ISCM OR heart failure OR ischemic cardiomyopathy OR ischemic cardiomyopathy).

### Investigating the Mode of Action Network of Candidate Drugs

In order to discover the potential MOA of candidate drugs, we built a MOA network for each candidate using a systems biology approach. The foundation of the MOA network is a previously described drug-disease network (DDN) (Peyvandipour et al., 2018) that considers the interactions between known drug targets and known HF-related genes. The construction of this MOA network was based on the HF-related network constructed in step 2 of our repositioning approach. Known HF-related genes extracted from Comparative Toxicogenomics Database were used to form the HF-related gene set as *S*_disease_ = (*x*_1_…*x*_n_). Then, given the set of targets of a certain drug that were involved in the disease network as *S*_drug_ = (*y*_1_…*y*_m_), we extracted DDN from the HF-related network as the MOA network of the drug. The DDN consists of all of the shortest paths in which a gene from either *S*_disease_ or *S*_drug_ can be a source or destination. To ensure the authenticity of the interaction between known drug targets and known HF-related genes, the steps from source to destination were limited to less than three.

### Discovering Potential MOAs Using KEGG Pathway Analysis

We used genes involved in each MOA network as an input to perform Kyoto Encyclopedia of Genes and Genomes (KEGG) pathway analysis to uncover potential pathways through which the candidate may perform its therapeutic efficiency to HF. The KEGG pathway analysis was performed using Enrichr (Chen et al., 2013), an integrative gene set enrichment tool used to perform pathway analysis. For each input gene list, the tool calculates an enrichment score based on a modified Fisher’s exact test. The adjusted *p* value cutoff was set to 0.05 for the identification of significant pathways. The significant pathways of each drug were ranked according to combined scores, and the HF-related pathways within the top 10 ranked pathways were selected as potential MOAs.

## Results

### Preliminary Candidates for HF

#### HF Arising From DCM

**Table 1** presents the selected top 5% (38) candidates for DCM. As expected, our analyses identified drugs used to treat HF in clinical practice, such as perindopril, telmisartan, and amiloride.

**Table 1:** Preliminary candidate drugs for DCM.

Class	Drug	Rank	Class	Drug	Rank
AI	nimesulide	1	ERA	estrone	20
ERA	estriol	2	CA	gemfibrozil	21
Other	tetrabenazine	3	NA	haloperidol	22
CA	**amiloride**	4	Other	metyrapone	23
CA	**atorvastatin**	5	CA	**nicardipine**	24
AI	azathioprine	6	Other	norgestimate	25
AI	clocortolone	7	CA	**perindopril**	26
ERA	dienestrol	8	CA	**pravastatin**	27
ERA	**estradiol**	9	Other	primaquine	28
Other	fulvestrant	10	CA	ritodrine	29
Other	letrozole	11	CA	**telmisartan**	30
Other	liothyronine	12	AAA	tripelennamine	31
NA	metixene	13	AAA	triprolidine	32
Other	**naloxone**	14	AAA	alimemazine	33
Other	progesterone	15	NA	amisulpride	34
Other	resorcinol	16	Other	atovaquone	35
NA	tranylcypromine	17	NA	**Bromocriptine**	36
Other	chloroquine	18	AI	budesonide	37
ERA	equilin	19	CA	**chlorpromazine**	38

Drugs whose names with bold font have literature support to be efficient to HF. CA, cardiovascular agent; ERA, estrogen receptor agonist; NA, neuropsychiatric agent; AI: anti-inflammatory; AAA, anti-allergic agent.

Besides, a total of 7 drugs from the list of 38 candidates had literature evidence supporting their use to treat HF. Animal studies have shown that estradiol treatment can prevent the development of HF (Beer et al., 2007; Satoh et al., 2007). Moreover, estradiol can also prevent cardiomyocyte apoptosis (Patten et al., 2004) and cardiac fibrosis (Pedram et al., 2010), which are involved in the pathological processes of HF. Chlorpromazine is a prototypical phenothiazine antipsychotic drug, and previous studies have shown that intravenous administration of chlorpromazine had hemodynamic effects and was of benefit to patients with HF (Elkayam et al., 1977; Mifune et al., 1979). Nicardipine is a calcium channel blockader exhibiting antihypertensive properties and is effective in the treatment of coronary spasms and angina. Studies showed that an acute intravenous drip infusion of nicardipine is effective in the treatment of HF (Fifer et al., 1990; Sy et al., 1993; Hirota et al., 1997). Naloxone is an opioid antagonist medication used to block the effects of opioid drugs. Animal experiments have shown that naloxone improves the systemic hemodynamics and myocardial contractile function in conscious dogs with HF (Himura et al., 1994). Atorvastatin and pravastatin are members of the drug class known as stains used to lower cholesterol. It has been suggested that the use of atorvastatin or pravastatin in HF patients attenuates adverse left ventricular remodeling and improves left ventricular systolic function as well as clinical outcomes (Sola et al., 2006; Wojnicz et al., 2006; Han et al., 2007; Lipinski et al., 2009; Correale et al., 2013). Bromocriptine is a prolactin release inhibitor. Bromocriptine treatment was reported to improve hemodynamic profiles in HF patients and was associated with a high rate of full left ventricular recovery and low morbidity and mortality in HF patients arising from peripartum cardiomyopathy (Francis et al., 1983; Hilfiker-Kleiner et al., 2017).

#### HF Arising From ISCM

**Table 2** presents the selected top 5% (38) of candidate drugs for ISCM. Amiloride was among the candidates. We also determined that 5 out of the 38 candidate drugs have been reported as potentially being useful in HF treatment. Estradiol, atorvastatin, and naloxone, which are candidate drugs of DCM, also appeared in the preliminary candidate drug list of ISCM. Preclinical and clinical studies have indicated that thalidomide, which displays immunosuppressive and antiangiogenic activity, has potential efficacy for HF patients (Agoston et al., 2002; Gullestad et al., 2002; Gullestad et al., 2005; Orea-Tejeda et al., 2007). Furthermore, studies have indicated that nitrendipine, a calcium channel blocker with marked vasodilator action, can favorably alter performance of the failing left ventricles in HF patients (Cohn, 1986).

**Table 2:** Preliminary candidate drugs for ISCM.

Class	Drug	Rank	Class	Drug	Rank
ERA	dienestrol	1	NA	citalopram	20
ERA	**estradiol**	2	AN	etoposide	21
Other	repaglinide	3	ANA	eugenol	22
Other	sulfinpyrazone	4	CA	flecainide	23
Other	tetracycline	5	NA	galantamine	24
Other	atovaquone	6	CA	gemfibrozil	25
Other	chloroquine	7	Other	itraconazole	26
ERA	equilin	8	CA	minoxidil	27
ERA	estrone	9	CA	**nitrendipine**	28
NA	ethotoin	10	CA	oxprenolol	29
CA	indapamide	11	NA	pentobarbital	30
NA	metixene	12	AN	sonidegib	31
ANA	**naloxone**	13	Other	triprolidine	32
ANA	naltrexone	14	Other	tropisetron	33
CA	nimodipine	15	CA	warfarin	34
Other	quinine	16	Other	albendazole	35
Other	**thalidomide**	17	CA	**amiloride**	36
NA	zonisamide	18	AN	amsacrine	37
CA	chlorthalidone	19	CA	**atorvastatin**	38

Drugs whose names with bold font have literature support to be efficient to HF. CA, cardiovascular agent; ERA, estrogen receptor agonist; NA, neuropsychiatric agent; AN, anti-neoplastic; ANA, analgesic.

### Refined List of Candidate Drugs

For DCM, 13 drugs remained after the second round of selection (**Table 3**). Perindopril and telmisartan were retained. In addition, five out of the aforementioned seven preliminary candidate drugs having been reported in the literature as having potential efficacy for HF treatment were also retained. For ISCM, 23 drugs remained following the second round of selection (**Table 4**). Amiloride and four out of five drugs supported by the literature in having potential therapeutic efficacy for HF were retained.

**Table 3:** Final candidate drugs for DCM.

Drug	Classification	Drug	Classification
**perindopril**	Cardiovascular agent	norgestimate	PR agonist
**telmisartan**	Cardiovascular agent	nimesulide	Anti-inflammatory
**nicardipine**	Cardiovascular agent	azathioprine	Anti-inflammatory
**chlorpromazine**	Cardiovascular agent	tranylcypromine	Neuropsychiatric
**estradiol**	ER agonist	**bromocriptine**	Neuropsychiatric
estrone	ER agonist	**naloxone**	Analgesic
progesterone	PR agonist		

Drugs with bold font have literature support to be efficient to HF.

**Table 4:** Final candidate drugs for ISCM.

Drug	Classification	Drug	Classification
nimodipine	Cardiovascular agent	dienestrol	ER agonist
flecainide	Cardiovascular agent	**estradiol**	ER agonist
gemfibrozil	Cardiovascular agent	equilin	ER agonist
**nitrendipine**	Cardiovascular agent	estrone	ER agonist
oxprenolol	Cardiovascular agent	**thalidomide**	Antineoplastic
**amiloride**	Cardiovascular agent	etoposide	Antineoplastic
metixene	Neuropsychiatrics	sonidegib	Antineoplastic
zonisamide	Neuropsychiatrics	amsacrine	Antineoplastic
galantamine	Neuropsychiatrics	repaglinide	Antidiabetic
pentobarbital	Neuropsychiatrics	quinine	Antimalarial
**naloxone**	Analgesic	albendazole	Anthelmintic
eugenol	Analgesic		

Drugs with bold font have literature support to be efficient to HF.

### Enrichment Analysis of Known HF Drugs

We extracted all drugs (21) being demonstrated as improving the prognosis of HF patients from the 762 drugs to perform the enrichment analysis. The 21 drugs include 9 angiotensin-converting enzyme inhibitors, 7 angiotensin II-receptor blockers, 2 aldosterone receptor antagonists, and 3 beta-blockers, metoprolol, bisoprolol, and carvedilol.

**Figure 2:**
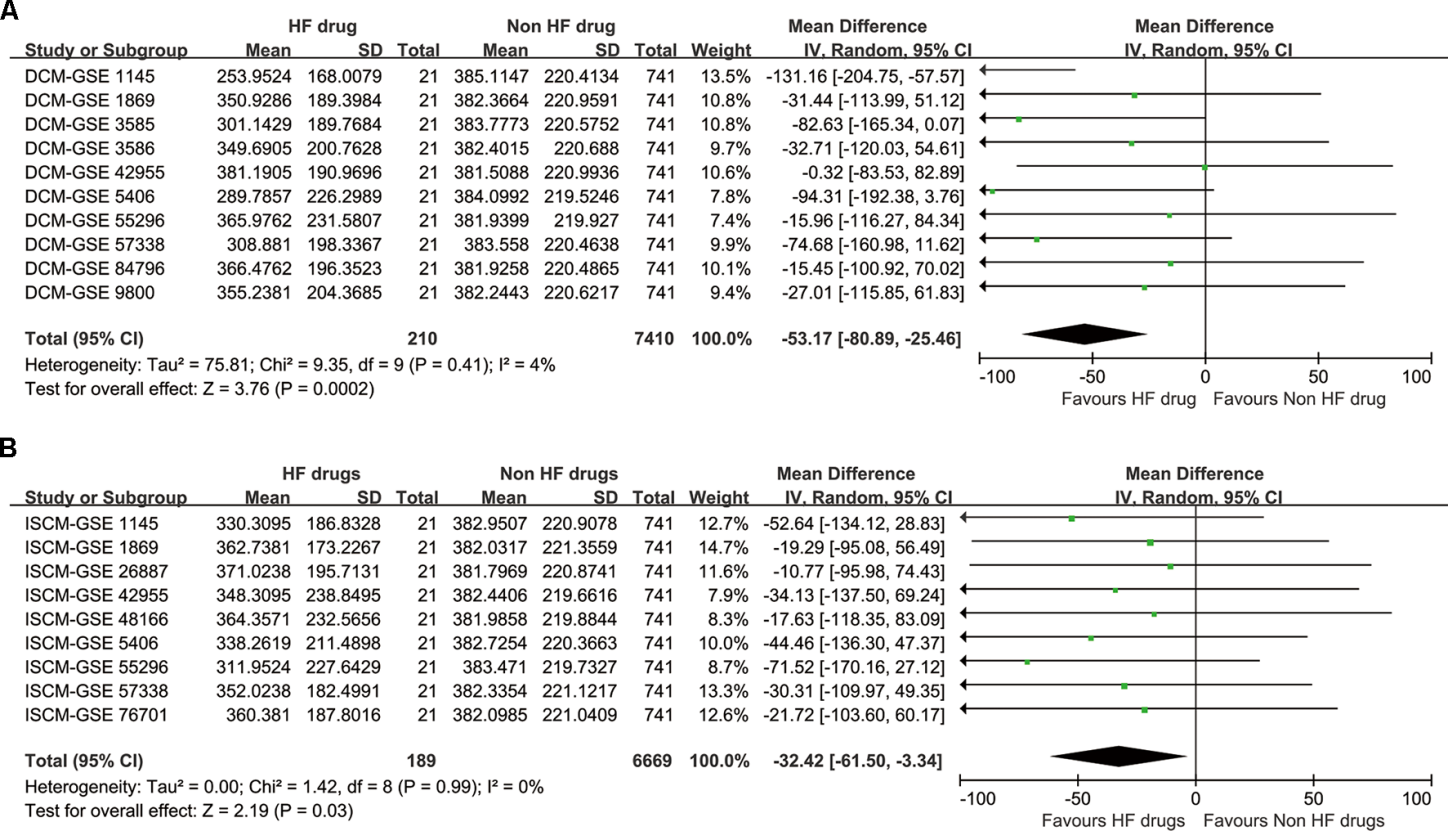
The synthesized result of the enrichment analysis. **(A)** For dilated cardiomyopathy (DCM). **(B)** For ischemic cardiomyopathy (ISCM).

Although Fisher’s methods and Tippett’s minimum P method only showed significant enrichment for DCM (**Table 5**), the inverse-variance method showed that the drug set was enriched for both DCM and ISCM, with significant *P* values of 0.0002 and 0.03, respectively (**Figure 2**). The forest plot also showed that the mean rank of the 21 drugs is smaller than that of the remaining 741 drugs in each data set without heterogeneity, indicating a robustness of the trend toward enrichment.

**Table 5:** Drug analysis enrichment synthesized P values.

	P value	
	DCM	ISCM
Fisher’s method	0.0326	0.6977
Toppett’s minimum p approach	0.0198	0.8131

### Potential MOA of Candidates

**Figure 3:**
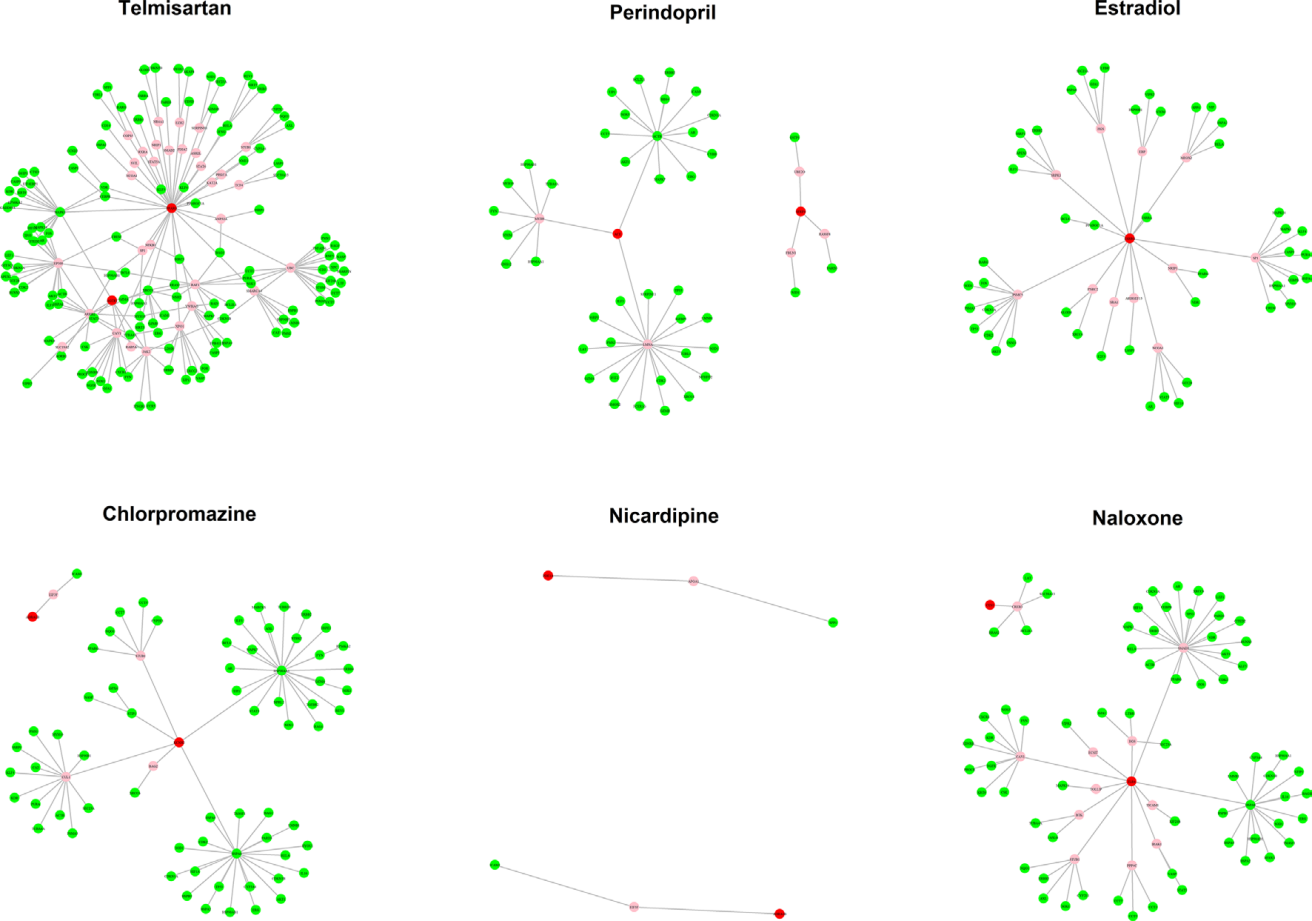
The mode of action (MOA) network of dilated cardiomyopathy (DCM) candidates. Green nodes present known heart failure (HF)-related genes. Red nodes represent drug targets. Pink nodes represent genes with no evidence of corresponding to HF.

**Figure 4:**
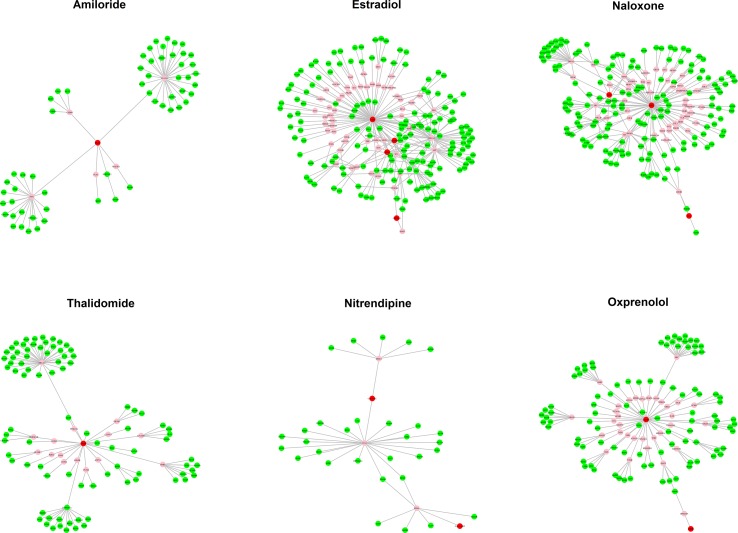
The mode of action (MOA) network of ischemic cardiomyopathy (ISCM) candidates. Green nodes present known heart failure (HF)-related genes. Red nodes represent drug targets. Pink nodes represent genes with no evidence of corresponding to HF.

The MOA networks displaying the relationship between drug targets and HF-related genes are shown in **Figures 3** and **4**. The significant pathways related to each MOA network are presented in **Supplemental Tables 3** and **4**.

#### Potential MOA of Candidates for DCM-Induced HF

Estradiol, chlorpromazine, nicardipine, naloxone, and bromocriptine were candidates with literature support regarding their potential efficacy for HF treatment. The enriched pathways using genes from their MOA network are described in the following section.

There are 59 genes contained in the MOA network of estradiol. Using these 59 genes as input for KEGG pathway analysis, the estrogen signaling pathway and hypoxia-inducible factor 1 (HIF-1) signaling pathway were deemed as significant. Notably, estrogen regulates a plethora of physiological processes in mammals through the estrogen signaling pathway, including prevention of the development of HF, cardiomyocyte apoptosis, and cardiac fibrosis (Patten et al., 2004; Beer et al., 2007; Satoh et al., 2007; Pedram et al., 2010). HIF-1 is a transcription factor functioning as a major regulator of oxygen homeostasis. One previous study investigated left ventricular tissues from HF patients and determined that the HIF pathway persists during HF (Zolk et al., 2008).

There are 72 genes in the MOA network of chlorpromazine. The significant pathways related to chlorpromazine include the phosphoinositide-3-kinase (PI3K)-Akt signaling pathway, the HIF-1 signaling pathway, and the mitogen-activated protein kinase *(MAPK)* signaling pathway. The PI3K-Akt signaling pathway regulates fundamental cellular functions such as transcription, translation, proliferation, growth, and survival. One study indicated that the PI3K-Akt signaling pathway is involved in the myocardial remodeling in HF patients and plays an important role in the pathogenesis of myocardial hypertrophy (Yang et al., 2005). The MAPK signaling pathway is a highly conserved module that is involved in the majority of cellular functions, including cell proliferation, differentiation, and migration. Previous studies have shown that downregulation of the MAPK signaling pathway has a cardioprotective effect against HF (Li et al., 2018).

The MOA network of nicardipine consists of six genes. The calcium signaling pathway is found to be related to the MOA network of nicardipine. Ca^2+^ plays an important role in connecting the excitability of cell membrane with contraction in myocardium. Previous reports have shown that Ca^2+^ homeostasis is defective in HF and represents a central cause of contractile dysfunction in failure myocardium (Luo and Anderson, 2013), suggesting that the calcium signaling pathway may be translated into novel therapies for HF.

There are 80 genes contained in the MOA network of naloxone. The PI3K-Akt signaling pathway and HIF-1 signaling pathway are significantly related to the MOA network of naloxone, which are both involved in the pathogenesis of HF (Yang et al., 2005; Zolk et al., 2008).

Although the MOA network of bromocriptine contains only three genes, the tumor necrosis factor (TNF) signaling pathway and nuclear factor kappa B (NF-kappa B) signaling pathway are deemed as significant. TNF can induce a wide range of intracellular signal pathways including apoptosis and cell survival as well as inflammation and immunity. Previous studies indicated that the expression of TNF receptors is upregulated in mononuclear leukocytes of HF patients (Nozaki et al., 1998). Besides, increased levels of TNF-alpha were observed in HF patients, which were high enough to reduce cardiac contractility *in vitro*, and the mortality of HF patients increases with higher levels of TNF-alpha (Muller-Ehmsen and Schwinger, 2004). For these reasons, TNF signaling pathway may appear to be a potential target for the treatment of HF. NF-kappa B is the generic name of the relevant transcription factor family. The NF-kappa B signaling pathway regulates genes involved in immunity, inflammation, and cell survival. The polymorphism of NF-kappa B is associated with the heart function of HF patients (Santos et al., 2010). Moreover, silencing the NF-kappa B signaling pathway can prevent cardiac hypertrophy and HF in mice HF models (Gupta et al., 2008).

Perindopril and telmisartan are currently being used in clinical practice to treat HF by targeting and inhibiting the renal–angiotensin–aldosterone system (RAAS). The significant HF-related pathways related to them include apoptosis, the PI3K-Akt signaling pathway, the HIF-1 signaling pathway, and the MAPK signaling pathway. Previous studies have revealed that cardiocyte apoptosis exists in the hearts of HF patients (Narula et al., 1996) and that the attenuation of apoptosis can delay cardiac dysfunction in murine HF models (Mandl et al., 2011). Moreover, high RAAS activity can result in apoptosis, while inhibiting RAAS can prevent apoptosis (Velez Rueda et al., 2012; Kooptiwut et al., 2015). Based on the literature, downregulating the MAPK signaling pathway can have cardioprotective effects against HF (Li et al., 2018), while inhibiting RAAS can abrogate MAPK activation (Lebeche et al., 2001).

Apart from the aforementioned drugs, other candidates were neither used in clinical practice nor did they exhibit any therapeutic efficacy to HF supported by the literature. However, there are at least two pathways corresponding to HF for each drug (results not shown), suggesting that these drugs may have potential therapeutic efficacy for HF treatment.

#### Potential MOA of Candidates for ISCM-Induced HF

Five candidate drugs of ISCM are used in clinical practice to treat HF or have literature support with known treatment efficacy for HF, including estradiol, naloxone, thalidomide, nitrendipine, and amiloride. Estradiol and naloxone are also DCM candidates, despite their MOA networks for DCM and ISCM being different. The MOA networks of estradiol and naloxone consist of 218 and 206 genes, respectively. The pathways that deemed as significant to them are the same, including the HIF-1 signaling pathway, MAPK signaling pathway, and PI3K-Akt signaling pathway. A total of 103 genes are involved in the MOA network of Thalidomide, and the HIF-1 signaling pathway and PI3K-Akt signaling pathway are related to this network. The MOA network of nitrendipine contains 35 genes, and the significant pathway of these genes is the PI3K-Akt signaling pathway. Amiloride, which is used as adjunctive treatment with diuretics in HF, was also contained in the final candidates list. We input the 63 genes of the MOA network of amiloride to perform the KEGG pathway analysis and determined that the HIF-1 signaling pathway, PI3K-Akt signaling pathway, and TNF signaling pathway were deemed significant, which suggests that amiloride may play other roles in the treatment of HF apart from diuretic activity. For the remaining drugs, at least two pathways corresponded to HF (results not shown), suggesting a potential therapeutic efficacy for HF prevention.

## Discussion

In the present work, we presented a two-step drug repositioning pipeline and applied our methodology to HF arising from two different etiologies to identify candidate drugs. Drug-set enrichment analysis and literature search provided support to the validity of our repositioning approach. MOA network and KEGG analysis displayed the potential mechanisms of drug efficacy.

Concerning the repositioning results, we observed that the cardiovascular drugs have the largest proportion among the candidates in both DCM- and ISCM-induced HF, indicating that our proposed method could specifically focus the repositioning on disease-related tissues. For DCM, the probability of finding candidates supported by literature rise from 26.3% (10 out of 38) to 53.8% (7 out of 13) after narrowing down the preliminary candidates, and this number rise from 15.8% (6 out of 38) to 21.7% (5 out of 23) for ISCM, which indicates that our two-step drug repositioning approach can effectively increase the likelihood of finding candidates with actual therapeutic potential for HF.

It is encouraging to know that our repositioning results are supported by drug-set enrichment analysis. Our results suggest that gene expression profiling data contains useful information for drug discovery. We observed that the inverse-variance method indicates that the drug set is enriched for both DCM and ISCM, while Fisher’s method and Tippett’s minimum P method only indicate significant enrichment for DCM. There are a few possible explanations for this result. First, the sample size of drugs used to do enrichment analysis may not be large enough. The number of drugs used to perform enrichment analysis is 21, which is much smaller than the total number of drugs used for drug repositioning (762). Second, HF is known to be heterogeneous with several subtypes, which may lead to different responses to the same therapeutic interventions (McMurray et al., 2012; Ahmad et al., 2014; Ahmad et al., 2018). Some studies have reported that ISCM-induced HF patients have a worse prognosis than DCM-induced HF patients (Follath et al., 1998; Follath, 2000), suggesting that the response to treatment in DCM-induced patients is better than that of ISCM-induced patients.

To investigate the MOA for the candidate drugs, we take advantage of existing information on known HF-related genes, known drug targets, and known protein–protein interaction information to obtain the MOA network for each drug. The MOA network displays the direct interactions between drugs and diseases in a form of network through its target genes. In the MOA network, we can identify genes that have not been previously reported as HF-related but have direct interactions with known HF-related genes as well as known drug targets, suggesting that these genes may represent potential biomarkers or therapeutic targets for HF. We also observe that, although some drugs appear in the candidates list of both DCM-induced and ISCM-induced HF, the MOA network can be different, which may indicate the differences of pathogenesis in HF arising from different etiologies. For candidates which are neither used in clinical practice nor supported by previous studies, HF-related pathways can be enriched using the genes contained in their MOA networks, indicating their potential therapeutic efficacy for HF.

There are a number of advantages to our study. First, we extracted all HF-related gene expression datasets with a minimum sample size of 10 from GEO and synthesized the results of each dataset to obtain a more robust and reliable results to compensate for the limitation that most human genomic studies of HF are limited by insufficient cardiac tissues. Second, the first step of our repositioning approach is a largely hypothesis-free approach blinding to any knowledge regarding existing HF drugs, known drug targets, or known drug–disease relationships, and thus is likely to find candidate drugs of different mechanisms from the known treatment. Third, compared to the Cmap methods used as the first step of our repositioning approach, the second step of our repositioning approach can effectively increase the likelihood of identifying candidates with actual therapeutic potential for HF and can be used in conjunction with any other drug repurposing methods to effectively narrow down a list of candidate drugs. In the second step, we determined the interaction between known drug targets and HF networks to narrow down the preliminary candidates. Nearly all preliminary candidate drugs being used in clinical practice or with literature support were included in the final candidates list. Fourth, our method of constructing MOA networks can be applied to any drug repositioning studies aimed at exploring the underlying mechanisms of action of candidate drugs. Fifth, to our knowledge, this is the first systematic drug repositioning analysis conducted in relation to HF. In the face of the growing epidemiological burden of HF, our study might be of particular use in the discovery of novel effective pharmacological therapies for HF.

The present study also has a few general limitations. First, although the pattern matching method used in the first step is largely hypothesis free, the assumptions of reversed expression patterns may not be completely true for every disease–drug pair. Second, in the second step of our repositioning approach, to ensure that drugs have the greatest likelihood to interact with HF, we only chose drugs whose targets are located in the HF network as the final candidates. Owing to this selection criterion, some candidates may be missed. Moreover, the drug-induced gene expression profiles in Cmap are not measured in cardiac tissues and may represent another limitation, although the Cmap study claimed that these data can be reasonably modeled in non-neoplastic diseases (Lamb et al., 2006). In addition, our repositioning analysis is only based on gene expression information. Considering that the gene expression profile only partly reflects the mechanisms of diseases and drugs, our method may be improved by incorporating other information related to genomics, transcriptomics, proteomics, and metabolomics. Finally, although we performed drug set enrichment analysis and literature search to validate our repositioning results and obtained promising results, the current study does not provide confirmatory evidence for the repositioning candidates, and thus, adequately sized preclinical and clinical studies will be necessary to verify the repositioning predictions.

In conclusion, we have proposed a framework for drug repositioning and presented a list of repositioning candidates for HF. To our knowledge, this is the first systematic drug repositioning analysis to HF, and we believe that it could be of particular use in the discovery of novel effective pharmacological therapies for HF.

## Data Availability

Publicly available datasets were analyzed in this study. This data can be found here: GSE57338, GSE5406, GSE1145, GSE55296, GSE3586, GSE3585, GSE84796, GSE42955, GSE1869, GSE9800, GSE76701, GSE48166, GSE26887, GSE92742, https://www.drugbank.ca/releases/latest, https://downloads.thebiogrid.org/BioGRID, http://ctdbase.org/downloads/#gd. 

## Author Contributions

GY, AM, and ZSQ conceived and designed the study. GY performed data analysis. GY, and ZSQ interpreted the data. GY and ZSQ wrote the manuscript. All authors approved the final version of the manuscript.

## Conflict of Interest Statement

The authors declare that the research was conducted in the absence of any commercial or financial relationships that could be construed as a potential conflict of interest.
