# Supplementary material for: An Integrated System Biology Approach Yields Drug Repositioning Candidates for the Treatment of Heart Failure
Source: Front Genet. 2019 Sep 25;10:916. doi: 10.3389/fgene.2019.00916 (PMC6773955; doi:10.3389/fgene.2019.00916)
Supplement: Supplementary file 1 [file Table_1.doc]

Supplemental Table 1 DCM datasets information

| Datasets | Platform | Sample.N | Sample.D |
| --- | --- | --- | --- |
| GSE 57338 | GPL 11532 | 136 | 82 |
| GSE 5406 | GPL 96 | 16 | 108 |
| GSE 1145 | GPL 570 | 11 | 27 |
| GSE 55296 | GPL 16288 | 10 | 13 |
| GSE3586 | GPL3050 | 15 | 13 |
| GSE3585 | GPL96 | 5 | 7 |
| GSE84796 | GPL14550 | 7 | 10 |
| GSE42955 | GPL6244 | 5 | 12 |
| GSE1869 | GPL96 | 6 | 6 |
| GSE9800 | GPL887 | 11 | 12 |

Sample.N: sample size of normal people. Sample. D: sample size of patients.

Supplemental Table 2 ISCM datasets information

| Datasets | Platform | Sample.N | Sample.D |
| --- | --- | --- | --- |
| GSE 57338 | GPL 11532 | 136 | 95 |
| GSE 5406 | GPL 96 | 16 | 86 |
| GSE 1145 | GPL 570 | 11 | 31 |
| GSE 55296 | GPL 16288 | 10 | 13 |
| GSE76701 | GPL570 | 4 | 4 |
| GSE42955 | GPL6244 | 5 | 12 |
| GSE48166 | GPL9115 | 15 | 15 |
| GSE1869 | GPL96 | 6 | 10 |
| GSE26887 | GPL6244 | 5 | 12 |

Sample.N: sample size of normal people. Sample. D: sample size of patients.

Supplemental Table 3 pathways related to the MOA network of DCM candidates supported by literature

| Term | P value | Adj  p value | Z score | Combined score | genes |
| --- | --- | --- | --- | --- | --- |
| **estradiol** |  |  |  |  |  |
| Estrogen signaling pathway | 4.87E-10 | 1.46E-08 | -1.812 | 38.845 | HSPA8;HSP90AA1;SP1;AKT1;MAPK1;HSPA2;FOS;HSP90B1 |
| HIF-1 signaling pathway | 6.7E-10 | 1.68E-08 | -1.709 | 36.097 | CDKN1A;ERBB2;STAT3;AKT1;MAPK1;LTBR;HIF1A;RELA |
| **Chlorpromazine** |  |  |  |  |  |
| PI3K-Akt signaling pathway | 3.06E-09 | 6.93E-08 | -1.911 | 37.476 | CDKN1A;HSP90AA1;CDKN1B;HSP90AB1;NOS3;CDK2;KDR;AKT1;SGK1;TP53;RELA;HSP90B1 |
| HIF-1 signaling pathway | 3.42E-09 | 6.93E-08 | -1.655 | 32.261 | CDKN1A;CDKN1B;NOS3;ERBB2;STAT3;AKT1;HIF1A;RELA |
| MAPK signaling pathway | 2.55E-08 | 4.59E-07 | -1.794 | 31.369 | HSPA8;NTRK2;MAPK7;RPS6KA2;AKT1;HSPB1;HSPA2;TP53;RELA;TGFBR2 |
| **nicardipine** |  |  |  |  |  |
| Calcium signaling pathway | 0.00118 | 0.019469 | -1.933 | 13.031 | PDE1A;ADRA1B |
| **naloxone** |  |  |  |  |  |
| PI3K-Akt signaling pathway | 2.24E-14 | 9.64E-13 | -1.989 | 62.521 | NGFR;CDKN1A;HSP90AA1;CDKN1B;HSP90AB1;NOS3;FASLG;RELA;CREB3;CDK2;KDR;AKT1;MAPK1;SGK1;TP53;TLR4;BCL2L1 |
| HIF-1 signaling pathway | 3.05E-13 | 7.5E-12 | -1.682 | 48.471 | CDKN1A;CDKN1B;NOS3;ERBB2;STAT3;AKT1;MAPK1;LTBR;HIF1A;TLR4;RELA |
| **Bromocriptine** |  |  |  |  |  |
| TNF signaling pathway | 0.01641 | 0.031219 | -1.699 | 6.9837 | ICAM1 |
| NF-kappa B signaling pathway | 0.013886 | 0.031219 | -1.597 | 6.8283 | ICAM1 |

Supplemental Table 4 pathways related to the MOA network of ISCM candidates supported by literature

| Term | P value | Adj  p value | Z score | Combined score | genes |
| --- | --- | --- | --- | --- | --- |
| **Estradiol** |  |  |  |  |  |
| HIF-1 signaling pathway | 2.55E-18 | 1.78E-16 | -1.790 | 72.494 | CDKN1A;CDKN1B;NOS3;IFNGR1;STAT3;CYBB;PIK3R1;HIF1A;RELA;IGF1R;VEGFA;IL6;NPPA;ERBB2;EIF4EBP1;AKT1;MAPK1;TIMP1;LTBR |
| MAPK signaling pathway | 6.75E-17 | 3.53E-15 | -1.921 | 71.541 | HSPB1;RELA;MAPK9;MAPK7;MYC;RPS6KA2;NTF3;RPS6KA1;AKT1;MAPK1;CD14;FLNC;NTRK2;MEF2C;CHUK;RRAS2;HSPA2;MAPK14;GNG12;GADD45G;TGFBR2;IL1B;DDIT3;MAPKAPK2;MAPT |
| PI3K-Akt signaling pathway | 8.06E-16 | 2.11E-14 | -1.886 | 65.530 | CDKN1A;CDKN1B;PTEN;PIK3R1;BRCA1;RELA;IGF1R;MYC;KDR;SPP1;EIF4EBP1;AKT1;MAPK1;YWHAH;NGFR;HSP90AA1;CHUK;NOS3;GNG12;VEGFA;IL6;GNB2;CDK2;DDIT4;MDM2;SGK1;BCL2L1 |
| **Naloxone** |  |  |  |  |  |
| HIF-1 signaling pathway | 1.29E-21 | 1.37E-19 | -1.816 | 87.375 | CDKN1A;CDKN1B;NOS3;IFNGR1;STAT3;CYBB;PIK3R1;HIF1A;RELA;IGF1R;VEGFA;IL6;NPPA;ERBB2;EIF4EBP1;AKT1;HMOX1;MAPK1;TIMP1;LTBR;TLR4 |
| PI3K-Akt signaling pathway | 1.95E-17 | 8.29E-16 | -1.963 | 75.546 | CDKN1A;CDKN1B;PTEN;PIK3R1;BRCA1;RELA;IGF1R;MYC;KDR;SPP1;EIF4EBP1;AKT1;MAPK1;NGFR;HSP90AA1;CHUK;NOS3;GNG12;VEGFA;CREB3;IL6;GNB2;CDK2;DDIT4;MDM2;SGK1;TLR4;BCL2L1 |
| MAPK signaling pathway | 2.23E-15 | 4.76E-14 | -1.769 | 59.666 | NTRK2;CHUK;RRAS2;HSPB1;HSPA2;MAPK14;GNG12;RELA;GADD45G;TGFBR2;MAPK9;MAPK7;MYC;IL1B;DDIT3;RPS6KA2;MAPKAPK2;RPS6KA1;AKT1;MAPK1;CD14;MAPT;FLNC |
| **Thalidomide** |  |  |  |  |  |
| HIF-1 signaling pathway | 1.87E-13 | 1.05E-11 | -1.790 | 52.449 | CDKN1A;CDKN1B;NOS3;ERBB2;STAT3;EIF4EBP1;AKT1;PIK3R3;MAPK1;HIF1A;RELA;VEGFA |
| PI3K-Akt signaling pathway | 2.11E-11 | 3.56E-10 | -1.834 | 45.078 | CDKN1A;HSP90AA1;CDKN1B;NOS3;PIK3R3;BRCA1;RELA;VEGFA;CDK2;DDIT4;KDR;EIF4EBP1;AKT1;MAPK1;GRB2;SGK1 |
| **Nitrendipine** |  |  |  |  |  |
| PI3K-Akt signaling pathway | 1.76E-06 | 3.64E-05 | -1.911 | 25.331 | CDKN1A;HSP90AA1;CDKN1B;CDK2;KDR;MAPK1;KRAS |
